# Xenobiotic Sensor- and Metabolism-Related Gene Variants in Environmental Sensitivity-Related Illnesses: A Survey on the Italian Population

**DOI:** 10.1155/2013/831969

**Published:** 2013-07-07

**Authors:** Daniela Caccamo, Eleonora Cesareo, Serena Mariani, Desanka Raskovic, Riccardo Ientile, Monica Currò, Liudmila Korkina, Chiara De Luca

**Affiliations:** ^1^Department of Biomedical Sciences and Morpho-Functional Imaging, Polyclinic University of Messina, 98125 Messina, Italy; ^2^Laboratory of Tissue Engineering and Skin Pathophysiology, Dermatology Institute (IDI IRCCS), Via Monti di Creta 104, 00167 Rome, Italy; ^3^2nd Dermatology Division, Dermatology Institute (IDI IRCCS), 00167 Rome, Italy

## Abstract

In the environmental sensitivity-related illnesses (SRIs), multiple chemical sensitivity (MCS), chronic fatigue syndrome (FCS), and fibromyalgia (FM), the search for genetic polymorphisms of phase I/II xenobiotic-metabolizing enzymes as suitable diagnostic biomarkers produced so far inconclusive results, due to patient heterogeneity, geographic/ethnic differences in genetic backgrounds, and different methodological approaches. Here, we compared the frequency of gene polymorphisms of selected cytochrome P450 (CYP) metabolizing enzymes and, for the first time, the frequency of the xenobiotic sensor Aryl hydrocarbon receptor (AHR) in the three cohorts of 156 diagnosed MCS, 94 suspected MCS, and 80 FM/FCS patients versus 113 healthy controls. We found significantly higher frequency of polymorphisms CYP2C9∗2, CYP2C9∗3, CYP2C19∗2, CYP2D6∗4 and CYP2D6∗41 in patients compared with controls. This confirms that these genetic variants represent a genetic risk factor for SRI. Moreover, the compound heterozygosity for CYP2C9∗2 and ∗3 variants was useful to discriminate between either MCS or FM/CFS versus SMCS, while the PM ∗41/∗41 genotype discriminated between MCS and either SMCS or FM/CFS. The compound heterozygosity for CYP2C9 ∗1/∗3 and CYP2D6 ∗1/∗4 differentiated MCS and SMCS cases from FM/CFS ones. Interestingly, despite the distribution of the AHR Arg554Lys variant did not result significantly different between SRI cases and controls, it resulted useful for the discrimination between MCS and SMCS cases when considered within haplotypes in combination with CYP2C19 ∗1/∗2 and CYP2D6 ∗1/∗4. Results allowed us to propose the genotyping for these specific CYP variants, together with the AHR Arg554Lys variant, as reliable, cost-effective genetic parameters to be included in the still undefined biomarkers' panel for laboratory diagnosis of the main types of environmental-borne SRI.

## 1. Introduction

In the last years, much attention has been paid to a variety of pathological conditions sharing the common feature of an aberrant response triggered by airborne or other routes of exposure to low doses of environmental pollutants or xenobiotics, such as chemicals, drugs, heavy metals, electromagnetic, or nuclear radiations, in concentrations far below average reference levels admitted for environmental toxicants [1–4]. Indeed, the World Health Organization has collectively defined these conditions as “idiopathic environmental intolerances”(IEIs), namely multiple chemical sensitivity (MCS), fibromyalgia (FM), chronic fatigue syndrome (CFS), dental amalgam disease, and others, among which the intolerances to microbial and environmental allergens or toxins, drugs, vaccines, specific foods, synthetic implants, and possibly new biomaterials are also included [5–7]. In view of the progressively increasing knowledge and awareness regarding these diseases, they are presently better described collectively as “sensitivity-related illnesses” (SRIs) [7, 8].

SRI symptoms appear mainly in adult life, with higher prevalence in women, although a growing number of pediatric cases have been recently registered, and evidence is accumulating for a role of *in-utero *sensitization [6, 9]. For the prototypical MCS, the disease onset is commonly self-reported as a single precipitating event following a severe intoxication or as a chronic exposure to lower doses of an environmental pollutant [10, 11], either in the occupational or in the domestic setting. Then, the intolerance becomes chronic, with symptoms elicited not only by the same incitant but also by different, multiple incitants, resolving at their removal. Challenges for establishing differential diagnostic criteria for various SRI lie in: (i) the absence of consensus on case definition in all SRI conditions, with the exception of FM [12]; (ii) the wide variety of multiorgan symptoms, including psychosomatic, neurological, memory loss, mood disorders, posttraumatic distress, chronic muscular fatigue, chronic bronchitis and asthma, eye-nose-throat, gastrointestinal, cardiac, and autoimmune disorders; (iii) the variable individual sensitivity and genetic predisposition; (iv) the absence of clearly defined pathogenic mechanisms; (v) the variety of potential triggers; (vi) the absence of dose-dependent responses, even after provocation; and (vii) common comorbidity features with known autoimmune diseases like systemic lupus erythematosus, rheumatoid arthritis, or vitiligo [13–18].

To date, the diagnosis of MCS is based on the compliance to Cullen's inclusion anamnestic criteria [19] and on the score resulting from the Quick Environmental Exposure and Sensitivity Inventory (QEESI) [20, 21]. QEESI is a multistep questionnaire determining the levels of sensitization to chemical environmental triggers, scoring the type, localization and severity of symptoms after exposure, and the life impact. It was first validated by Miller and Prihoda (1999) [9] in the USA, adapted on the German population by Fabig (2000) [21], and successively validated in different national contexts, Japan, Sweden, Denmark [8], and, more recently, also in Spain [22].

The classification as “idiopathic” for these environmental intolerances arises from the poor current knowledge of their etiology and pathogenesis and the absence of recognized genetic and metabolic markers [8]. However, recent advances in toxicogenomics and metabolomics have highlighted the role of inherited or acquired impairment of xenobiotic metabolism in the individual hypersensitivity to both xenobiotics and toxic endogenous metabolites [14, 23–25]. Alterations of this system might lead to incomplete detoxification of exogenous/endogenous toxins or/and to excessive generation of toxic by-products, in the case of “poor metabolizers” (PM) or to higher-than-normal rates of metabolization in subjects with hyperfunctional genes, possibly duplicated or multiplicated, termed as “extensive metabolizers” (EMs) [11, 17, 26].

Leading studies on chemically hypersensitive individuals in European and North American populations have been so far focused on single-nucleotide polymorphisms (SNPs) in genes coding for a variety of phase I and phase II metabolizing enzymes and their receptors [17, 25, 27–32]. However, the conclusions obtained were limited and contradictory, mainly due to inhomogeneous and insufficient size of the patient and control groups and due to different and non standardized patient cohort inclusion criteria adopted by the different studies. Thus, the search for genetic markers distinctive of SRI remains under question, awaiting for conclusive evidence of their diagnostic value and cost-effectiveness.

Moreover, the identification of genetic markers specific for different SRI would be extremely useful for a better diagnosis assessment of MCS, FM, CFS and other idiopathic environmental intolerances. this work was aimed at assessing the distribution of some selected gene polymorphisms in representative groups of Italian hypersensitive individuals fully or partially diagnosed with MCS, or affected by its more common comorbidities, FM or FCS, as compared with sex- and age-matched healthy controls.

In these groups, we analyzed the frequency of some well-known polymorphic variants of cytochrome P450 (CYPs) enzymes, namely, (CYP2C9, CYP2C19, and CYP2D6), commonly investigated in different clinical conditions including MCS [29, 33, 34]. CYP family of phase I enzymes is essential for drug metabolism and bioactivation [35]. We then analyzed for the first time, in the same cohorts, the frequency of selected gene polymorphisms of the ligand-activated transcription factor Aryl hydrocarbon receptor (AHR) [36]. AHR was first known as the main controller of the expression of several classes of xenobiotic metabolizing enzymes (XMEs) [37] and then, more extensively, of a wide array of cell responses to organic chemical xenobiotic and UV environmental stressors [38] through multiple signal transduction pathways (recently reviewed in [39]).

Because of their critical role in the xenobiotic-induced toxicity, the potentially wide array of altered gene polymorphisms of the complex drug-metabolizing network in individuals affected by environmental intolerances so far not conclusively demonstrated in spite of rather extensive investigations, still remains of utmost interest for patient management and possibly also for therapeutic approaches.

## 2. Methods

### 2.1. Patients

Patients of case cohorts were selected on the basis of their diagnosis made according to Cullen's criteria [19] and QEESI questionnaire [21]. A modified QEESI score of 10 common environmental exposures and 10 major symptoms allowed to diagnose MCS (20 ≤ score ≤ 30) or SMCS (suspected MCS) (10 ≤ score ≤ 20) or others to be excluded from enrollment (0 ≤ score ≤ 10) [8].

One hundred and fifty-six Italian consecutive MCS patients (87 F/14 M, 49 ± 11 years), 94 consecutive SMCS patients (79 F/15 M; 49 ± 12 years), partly corresponding to the diagnostic criteria reported above, and 80 consecutive patients (61 F/19 M; 47 ± 10 years) presenting either fibromyalgia or chronic fatigue syndrome (FM/CFS group), were enrolled for this study by specialized clinicians, who performed anamnestic and lifestyle data collection, at Istituto Dermopatico dell'Immacolata, IRCCS, according to a study protocol approved by IDI Ethical Committee (no. 121/CE/2008).

One hundred and thirteen healthy Italian subjects (90 F/23 M, 53 ± 12) matched for sex and age with patient group were selected as controls (Ctr) at Istituto Dermopatico dell'Immacolata (IDI IRCCS) and Messina University according to the established criteria of (i) an absence of any clinically diagnosed disease, in particular allergic or immunologic disturbances, and (ii) no drug or nutraceutical supplement since at least six weeks at the time of blood sampling, (iii) whole blood total production of reactive oxygen and nitrogen species (ROS/RNS) below 650 cps/*μ*L, as determined by luminol-dependent chemiluminescent response to phorbol 12-myristate 13-acetate (PMA) [40] (IDI study protocol approval no. 52/CE/2010). Nonsmokers in the patient group were 70.4% (MCS), 82.2% (SMCS), and 84.2% (FM/FCS); smokers were 8.2% (MCS), 17.8% (SMCS), and 12.0% (FM/FCS). Patients with undetermined smoking habits were 21.4% (MCS) 0% (SMCS), and 3.8% (FM/FCS). Nonsmokers in the Ctr group were 85.2%. No alcohol or drug abusers were present neither in patients' nor in control groups.

Patients and controls were selected from different Italian regions in order to partially overcome the historical genetic variability in this country [41].

All subjects provided written informed consent to blood sampling and anamnestic data collection.

### 2.2. Genotyping for SNPs in Drug Metabolism-Related Enzymes

Genomic DNA was purified from 400 *μ*L of human whole blood using the QIAamp DNA Blood Mini Kit (Qiagen, Hilden, Germany) according to the manufacturer's instructions. DNA was quantified spectrophotometrically at 260 nm, aliquoted, and stored at −20°C until assayed.

Genotyping of SRI patients and control subjects for eight single nucleotide polymorphisms in drug metabolism- and inflammation-related genes was carried out by real-time PCR allelic discrimination using predesigned TaqMan SNP genotyping assays available from Applied Biosystems (Applera Italia, Monza, Italy). The polymorphisms analyzed were those of genes coding for: cytochrome P450 (CYP), family 2, subfamily C, polypeptide 9 and 19, namely, CYP2C9*2 (C > T, rs1799853; assay ID: C_25625805_10), CYP2C9*3 (A > C, rs1057910; assay ID: C_27104892_10), and CYP2C19*2 (G > A, rs4244285; assay ID: C_25986767_70); CYP2 subfamily D, polypeptide 6, namely, CYP2D6*4 (1846G > A, rs3892097; assay ID: C_27102431_D0) and CYP2D6*41 (C > T, rs28371725; assay ID: C_34816116_20); and aryl hydrocarbon receptor (AHR) Arg554Lys variant (G > A, rs2066853; assay ID: C_11170747_20).

Genotyping reactions were set up in a 96-well plate on a 7900HT Fast Real-Time PCR System (Applied Biosystems, Foster City, CA), and were carried out in a final volume of 20 *μ*L containing 1x TaqMan Genotyping Master Mix, 1x TaqMan-specific assay, and 10 ng genomic DNA, using thermal cycling conditions suggested by manufacturer's protocols.

### 2.3. Statistical Analysis

Allele and genotype frequencies obtained on patients' and control groups were compared by Fisher test analysis. Software employed was GraphPad Prism 4 (San Diego, CA, USA). Values of *P* ≤ 0.05 were considered as significant.

## 3. Results

**Figure 1:**
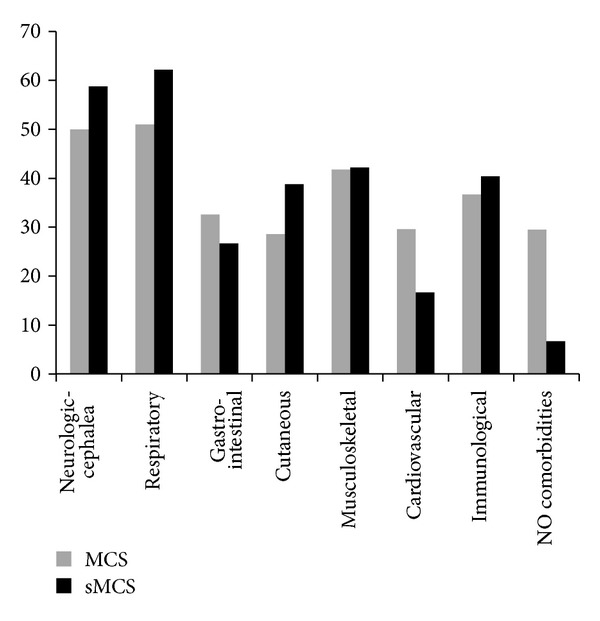
Comorbidities registered in the MCS group (*n* = 156; BMI = 23.4 mean ± 5.4 SD) and the sMCS group (*n* = 94; BMI = 23.8 mean ± 4.8 S.D.) through evaluation of anamnesis. Data are expressed as percentage of patients affected by each single category of organ pathologies. Abbreviations: body mass index (BMI); multiple chemical sensitivity (MCS); and suspected multiple chemical sensitivity (sMCS).

The main categories of co-morbidities resulting from anamnestic analysis are compared in MCS and SMCS patients and reported in Figure 1.

Genotype and allele frequencies of selected polymorphisms in drug metabolism-related genes examined in SRI patients (MCS, SMCS, and FM/CFS) and controls are shown in Table 1. Genotype frequencies among cases and controls were in Hardy-Weinberg equilibrium, and, similarly, allele frequencies among cases and controls were within the 95% confidence.

### 3.1. Analysis of CYP2C9 Genetic Background and Risk for Disease

The analysis of the distribution of CYP2C9 gene variants showed that the allele CYP2C9*2 was more present than the CYP2C9*3 both in MCS as well as in SMCS patients and in healthy subjects but not in FM/CFS group. However, both mutated alleles were more frequent in cases than in controls as the prevalence of CYP2C9*2 was around 1.5-fold higher in patients than in controls and that of CYP2C9*3 was 7-fold, 11-fold, and 21-fold higher in MCS, SMCS, and FM/CFS patients, respectively, than in controls (Table 1). However, significant differences were found only for the frequency of *2 allele between controls and other patients and for the frequency of *3 allele among all cases and controls and also between MCS patients and FM/CFS ones, as well as SMCS and FM/CFS patients (Table 1). Both CYP2C9*2 and CYP2C9*3 alleles were almost entirely represented by the heterozygous genotype, given that poor metabolizer homozygous individuals, either PM *2/*2 or PM *3/*3, were very few in each subgroup of patients and almost absent in the control population. The frequency of the CYP2C9*2 heterozygous genotype (Ht *1/*2) was higher in the MCS subgroup than in all other groups but reached a statistically significant difference only in comparison with control group. Notably, the frequency of the CYP2C9*3 heterozygous genotype (Ht *1/*3) was significantly higher in all groups of patients than in controls and in the group of FM/CFS patients compared with MCS one. Interestingly, compound heterozygotes for both CYP2C9 gene variants (PM *2/*3) were generally poorly represented in cases and absent in the SMCS subgroup and in healthy subjects (Table 1).

The calculation of odds ratio (ORs) showed that the presence of genotype Ht *1/*2 increases 2.4-fold the risk for developing MCS, while having the genotype Ht *1/*3 causes a 7.5-, 12-, and 26-fold increase of the risk for developing MCS, SMCS, and FM/CFS, respectively.

### 3.2. Analysis of CYP2C19 Genetic Background and Risk for Disease

Genotyping for the CYP2C19 gene variants showed that the frequency of the mutated *2 allele was significantly higher in MCS cases than in all other groups. Interestingly, the lowest frequency for this allele was observed in SMCS cases; moreover, healthy subjects and FM/CFS patients had a similar *2 allele frequency (Table 1). The CYP2C19*2 allele was almost entirely represented by the heterozygous genotype in all sampled subgroups and showed a significantly higher prevalence in MCS cases than in all other groups, while the frequency of homozygotes PM *2/*2 was similar among all subgroups (Table 1).

OR calculation showed that a mutated CYP2C19* background was associated with a threefold increased risk for developing MCS.

### 3.3. Analysis of CYP2D6 Genetic Background and Risk for Disease

The analysis of genetic background at the CYP2D6 gene locus showed that the CYP2D6*4 was the most represented gene variant in all subgroups of SRI cases compared with the CYP2D6*41; moreover, this latter variant was absent in the control population.

The comparison of allele frequencies showed that CYP2D6*4 allele had a highly significant, different frequency among all cases compared with controls, being two-threefold higher in patients than in healthy subjects. The CYP2D6*41 allele, that was absent in control population, had the highest frequency in FM/CFS group.

Both CYP2D6*4 and CYP2D6*41 alleles were largely represented by heterozygous individuals but displayed a different variability among the sampled subgroups. The CYP2D6 *1/*4 heterozygous genotype was more frequent in MCS cases than in either SMCS or FM/CFS patients and controls. However, the frequencies of Ht *1/*4 genotype in all three patient groups resulted significantly higher than in healthy subjects. Poor metabolizers, homozygotes *4/*4 were more frequent in the FM/CFS patients than in all other groups (Table 1), showing nonsignificant differences among them and tended to statistically significant difference (*P* = 0.06) in comparison with controls.

The CYP2D6 *1/*41 heterozygous genotype had a higher frequency in MCS group than in the other patients, having a similar distribution, and was absent in the control population, reaching a statistically significant difference. Poor metabolizers, compound heterozygotes *4/*41, and homozygotes *41/*41 were more frequent in FM/CFS patients than in SMCS as well as MCS cases, these latter lacking of the PM *41/*41 genotype, and both were absent in the control population (Table 1).

The calculation of odds ratio indicated that a genetic background positive for the presence of genotype CYP2D6 Ht *1/*4 increased three-fold and two-fold the risk for developing MCS and either SMCS or FM/CFS, respectively, while the same risk was 52-fold, 38-fold, and 36-fold increased by the presence of CYP2D6 Ht *1/*41.

### 3.4. Analysis of AHR Genetic Background and Risk for Disease

Genotyping for the Arg554Lys variant of AHR gene showed for the first time that the Lys-mutated variant was less frequent in MCS and SMCS cases, displaying similar distributions, than in FM/CFS patients and controls, and was entirely represented by the heterozygous genotype in all subgroups of cases, while the control population included both heterozygotes and homozygotes. Moreover, the frequency of heterozygotes was higher in the FM/CFS patients than in MCS, SMCS, and control groups, all showing similar frequencies (Table 2). However, these differences did not reach statistical significance.

### 3.5. Analysis of Haplotype Distribution

The analysis of the distribution of two or more combined mutant alleles, that is, the frequency of given haplotypes in the various sampled population, is reported in Table 3. It was shown that very few individuals were carriers of more than one mutation in the examined CYP isoforms. This was true both considering the occurrence of more than one mutation in the same gene, that is, the presence of both CYP2C9 variants and both CYP2D6 variants, and the occurrence of more than one mutation in different genes.

The most frequent haplotype in MCS cases was the CYP2C19 Ht *1/*2 - CYP2D6 Ht *1/*4- AHR Arg554Lys (9.1%), that was not present neither in SMCS cases nor in other patients (*P* < 0.001).

In the SMCS cases the most represented haplotype was the CYP2C9 Ht *1/*2-CYP2D6 Ht *1/*4, that was absent in the group of other patients and had a significantly lower frequency in MCS (*P* < 0.05).

In the subgroup of FM/CFS patients the most frequent haplotype was the CYP2C19 Ht *1/*2 - CYP2D6 Ht *1/*4, that showed a significantly higher distribution compared with SMCS cases (*P* < 0.01). Moreover, the haplotype CYP2C9 Ht *1/*3 - CYP2D6 Ht *1/*4 was only represented in MCS cases and SMCS, while the haplotype CYP2C9 Ht *1/*3- CYP2D6 Ht *1/*4 was only present in MCS cases. On the contrary, the combination of four mutated alleles either in heterozygosis or homozygosis was only observed in the subgroup of FM/CFS patients.

## 4. Discussion

To date a commonly agreed-upon set of laboratory metabolic parameters to be used worldwide for the classification of environmental-borne multiorgan syndromes, such as multiple chemical sensitivity is not available [6, 42]. Based on a consistent body of data on oxidative stress markers in fibromyalgia and chronic fatigue syndrome [8], previous reports by our research group have highlighted the validity, for the laboratory confirmation of clinical MCS diagnosis of an extensive biochemical characterization based on metabolic and immunological markers. These include alterations of erythrocyte catalase, glutathione peroxidase and transferase activities, glutathione depletion, and the polyunsaturated fatty acid-depleted profile of erythrocyte membrane, combined with specific plasmatic patterns of proinflammatory cytokine alterations [17, 25]. However, despite these available biological profiles, a reference panel useful for the unequivocal laboratory discrimination of MCS from other SRI, such as FM, CFS or for the confirmation of suspected MCS is still under construction.

Until now, the search for SRI biomarkers of disease has been centered on genetic determinants, including polymorphic variants of phase I/phase II detoxification enzymes, mainly analyzed in MCS subjects, with the study of a variety of cytochrome P450 isoenzymes (CYPs), glutathione-S-transferases (GSTs), UDP-glucuronosyl transferases (UGTs), catechol-O-methyltransferases (COMTs), N-acetyl transferases (NATs), paraoxonase 1 and 2 (PON1, PON2), methylenetetrahydrofolate reductase (MTHFR), and of cholecystokinin 2 receptor (CCKR2) [25, 27–32], although data are available also for FM [34, 43] and CFS [44].

Results, as a whole, have not been encouraging, given that the complexity and high costs of the investigations in that contrasting results were reported by different groups and the differences in genotype distribution between cases and controls were often not reaching statistical significance. However, a reliable between-groups comparison of outcomes from these studies was hard, due to the lack of homogeneity with regard to sample size, gender, and ethno-geographical features, of case/control populations examined. Furthermore, in general, positive findings from genetic association studies seem to be difficult to replicate and possibly inflated by chance [45].

Another critical issue is the heterogeneity of SRI, most of which exhibit overlapping symptoms with other common pathological conditions. Indeed, a diagnostic panel including defined criteria for diagnosis assessment, would be very important for etiological studies. Even though the MCS case definitions and the grading of chemical sensitivity in general populations used in the previous studies cited appear to be very similar, the underlying classification differences cannot be ruled out as they contribute to the inconsistency among published studies. In this regard, all patients included in our study have been diagnosed consecutively according to Cullen's criteria and adapted QEESI questionnaire scoring [19, 20, 25], by the same team of specialists, thus minimizing selection bias in the recruiting of patients and securing the uniform group as much possible.

Importantly, an additional critical issue is the use of genotyping techniques having 100% sensitivity and specificity for the detection of heterozygous genotypes, the most common form by which a mutant allele is present in a given population. In this regard, most of the published genetic investigations on MCS patient, including our previous report on 110 Italian subjects [25], employed the PCR-RFLP technique which, lacking of the required sensitivity, often provides a misrepresentation of genotype distribution in the population, specifically with regard to the frequency of heterozygotes [46].

Here, we investigated the distribution of some gene variants of clinically relevant drug metabolism-related proteins, that is, selected CYP enzyme isoforms, namely, CYP2C9*2, CYP2C9*3, CYP2C19*2, CYP2D6*4, and CYP2D6*41 already addressed in previous studies producing conflicting data. We reported here for the first time that the examined variants of CYP isoforms have a significantly different distribution in SRI patients versus controls. Most importantly, some of these variants may be useful for an objectively based discrimination between different types of SRI, given the observed significant differences after between-groups comparison of allele and genotype frequencies. In particular, allele and heterozygous genotype frequencies observed in all subgroups of SRI patients for the variant CYP2C9*3 showed significant or even highly significant differences with those observed in healthy subjects. These differences allowed us to propose the presence of the genetic variant CYP2C9*3 as a candidate marker of disease state with specific reference to SRI, as also confirmed by calculation of disease risk by Odds Ratio (OR), that was increased in a range from 7- to 26-fold for MCS, SMCS, and FM/CFS conditions, respectively. In particular, highly discriminant between pathological condition and healthy state was the presence of the homozygous genotype PM *3/*3 and the compound heterozygous one PM *2/*3 that was not recorded in control subjects. Moreover, the presence of the heterozygous genotype Ht *1/*3 may be useful for discrimination between MCS and FM/CFS patients, as the difference in genotype frequencies are highly significant.

The examination of genetic background for CYP2C9*2 showed that the allele *2 frequency may discriminate MCS patients and SMCS patients from FM/CFS ones and that the frequency of genotype Ht*1/*2 differentiates MCS patients from controls. As a whole, the search for this CYP2C9 variant may be quite useful in population-based studies for the classification of different SRI.

Similar results were obtained after comparison of allele and genotype frequencies for CYP2D6 variants in patients and controls, even though variable significance level for differences wre found. Both allele and heterozygous genotype frequencies for CYP2D6*4 and *41 resulted useful for discriminating between intolerance syndromes and healthy state. In particular, a genetic background positive for the presence of either genotype CYP2D6 Ht *1/*4 or Ht *1/*41 increased up to 3-fold and 50-fold the risk for developing SRI (see Table 1). Notably, the presence of poor metabolizer individuals, having either the homozygous genotype PM *41/*41 or the compound heterozygous one PM *4/*41, was not recorded in control subjects. These results, at least those demonstrating the association of CYP2D6 genetic variants with MCS state, are in agreement with those reported in a previous study on a Canadian population [29], where a relationship was found between MCS and CYP2D6 activity, with an OR of 2.49 for intermediate metabolizers and 3.36 for extensive metabolizers, while ultrarapid metabolizers were not examined. Finally, neither allele or genotype frequencies for both CYP2D6 variants may be regarded as differential markers between various disease states, given the observed between-groups similarities.

Interestingly, the analysis of allele and genotype distribution for the CYP2C19 variants showed that the examination of genetic background for this CYP isoform may be highly useful to diagnose MCS patients compared with healthy subjects, since individuals carrier of the heterozygous genotype CYP2C19 Ht *1/*2 has a threefold increased risk of developing MCS. Most importantly, the presence of genotype Ht *1/*2 is highly discriminant between MCS and either SMCS or FM/CFS patients.

Allele and genotype distribution for different CYP2C9 and CYP2D6 variants as well as for the CYP2C19 variant in MCS patients and healthy subjects recruited for this study resulted different, though not statistically significant from previously reported frequencies in Italian MCS patients by our group [25] and from both Caucasian and Italian general populations [26, 47]. In general, this may be prevalently due to the different technical approaches employed, since previous studies were based on the use of less sensitive PCR-RFLP technique. Additionally, and specifically with regard to the population of the affected individuals, the different frequencies observed in our previous study [25] could be derived from grouping together MCS individuals affected by different types of SRI comorbidities, including FM and CFS. Indeed, results obtained in the present study and discussed above, clearly show that allele and genotype frequencies for CYP2C9 variants as well as for the other examined CYP variants, are variable and reach statistically significant differences between the various disease states (MCS, SMCS, and FM/CFS). Thus, putting together affected individuals exhibiting different genetic backgrounds in one “disease” group may have generated a bias in those previously carried-out genetic studies.

Finally, some gene variants, both single or combined together, may be useful for the discrimination between different types of SRI or for the confirmation of clinically suspected MCS cases. In particular, the compound heterozygosity for CYP2C9 *2 and *3 variants may discriminate between the MCS and “other patients” groups versus SMCS, and the PM *41/*41 genotype may discriminate between MCS versus SMCS as well as versus patients with other pathologies. The compound heterozygosity for CYP2C9 Ht *1/*3 and CYP2D6 Ht *1/*4 may discriminate MCS cases as well as SMCS versus patients with other pathologies.

As a new direction of investigation, we here inquired if a described gene polymorphism of the transcription factor AHR - AHR Arg554Lys may be present with significant frequencies in a population of patients affected by specific SRI categories, fully diagnosed MCS, suspected MCS, and the FM/CFS group, in comparison with healthy individuals. Interestingly, although the distribution of the AHR Arg554Lys gene variant did not result significantly different between SRI cases and controls (Table 2), it proved indeed to be useful for the between-groups discrimination, when considered within different haplotypes (Table 3). In particular, we found that the haplotype CYP2C19 Ht*1/*2 - CYP2D6 Ht *1/*4- AHR Arg554Lys was only present in MCS cases, and the presence of the AHR variant was useful to differentiate MCS cases from SMCS patients, who showed also a high frequency of the haplotype CYP2C9 Ht *1/*2-CYP2D6 Ht *1/*4; this latter is absent in the group of “other patients.” On the contrary, the various combinations of mutant alleles of the three CYP isoforms with heterozygosity for the AHR Arg554Lys gene variant were only observed in the subgroup of the “other patients.”

In conclusion, on the basis of these observations, we propose the genotyping for CYP2C9*2 and *3, CYP2C19*2, and CYP2D6 *4 and *41 variants, together with the AHR Arg554Lys variant to be included in the panel of diagnostic biomarkers under construction aimed at the laboratory diagnosis of SRI and at the discrimination of the different specific conditions grouped under the collective definitions of “environmental-borne sensitivity-related illnesses,” more largely known as “idiopathic environmental intolerances”.

A further step of our study design will be the correlation between a given genotype and the corresponding biochemical profile as described by De Luca et al. [25], both to be also carefully correlated with assessed individual exposure levels to chemical toxicants, substrate of the examined receptor/enzyme-metabolizing actions. Given the gene-environment interactions and the fact of depending on the type of chemical toxicant and its metabolites, a given metabolizer phenotype may either confer protection or increase the risk of harmful effects; ruling out the effects of genetic background on the hypersensitive phenotype is expected to be very useful for clinical management.

## Figures and Tables

**Table 1:** Allele and genotype frequencies of some drug metabolism-related gene variants in SRI patients (MCS: multiple chemical sensitivity; sMCS: suspected multiple chemical sensitivity; FM/CFS: fibromyalgia/chronic fatigue syndrome) and healthy subjects (Ctr: controls).

Genotype	MCS	SMCS	FM/CFS	Ctr
CYP2C9	*N* = 101	*N* = 94	*N* = 80	*N* = 90

Wt	66.3%	66%	55%	84.4%
Ht*1/*2	23.8%^†^	18.1%	15%	12.2%
Ht*1/*3	6.9%^†,§§^	11.7%^††^	20%^††††^	1.1%
PM*2/*2	0.9%	2.1%	3.75%	2.2%
PM*2/*3	0.9%	—	2.5%	—
PM*3/*3	0.9%	2.1%	3.75%	—
*2 allele frequency	0.132^§§^	0.111^§^	0.125^†^	0.083
*3 allele frequency	0.048^††^	0.079^†††^	0.15^††††^	0.007

CYP2C19	*N* = 101	*N* = 93	*N* = 80	*N* = 62

Wt	61.4%	84.8%	78.8%	80.3%
Ht*1/*2	36.6%^††,###,§^	14.1%	20.0%	16.7%
PM*2/*2	2.0%	1.1%	1.3%	3.0%
*2 allele frequency	0.203^†,§,##^	0.081	0.113	0.113

CYP2D6	*N* = 55	*N* = 93	*N* = 75	*N* = 113

Wt	40%	52.7%	40.7%	77.9%
Ht*1/*4	36.4%^††^	25.8%^†^	28.5%^†^	18.6%
Ht*1/*41	14.8%^††††^	5.4%^††††^	7.4%^††††^	—
PM*4/*4	5.5%	4.3%	8.5%	3.5%
PM*4/*41	3.6%	7.5%	7.4%	—
PM*41/*41	—	4.3%	7.4%	—
*4 allele frequency	0.255^†††^	0.209^††^	0.314^††††^	0.128
*41 allele frequency	0.092^††††^	0.107^††††^	0.148^††††^	—

^†^ *P* < 0.05 versus Ctr, ^††^ *P* < 0.01 versus Ctr; ^†††^ *P* < 0.001 versus Ctr, ^††††^ *P* < 0.0001 versus Ctr;**** ^§^ *P* < 0.05 versus FM/CFS;**** ^§§^ *P* < 0.01 versus FM/CFS; ^#^ *P* < 0.05 versus SMCS; ^##^ *P* < 0.01 versus SMCS; ^###^ *P* < 0.001 versus SMCS.

**Table 2:** Allele and genotype frequencies of AhR-related gene variants in SRI patients (MCS: multiple chemical sensitivity; sMCS: suspected multiple chemical sensitivity; FM/CFS: $fibromyalgia/chronic fatigue syndrome) and healthy subjects (Ctr: controls).

Genotype	MCS	SMCS	FM/CFS	Ctr
AHR	*N* = 156	*N* = 56	*N* = 32	*N* = 54

Arg554Arg	80.1%	82.1%	68.7%	77.8%
Arg554Lys	19.9%	17.9%	31.3%	16.7%
Lys554Lys	—	—	—	5.6%
*A allele frequency *	0.099	0.089	0.156	0.139

**Table 3:** Haplotype frequencies of CYP2C9, CYP2C19, CYP2D6 and AHR gene variants in SRI patients (MCS: multiple chemical sensitivity; Smcs: suspected multiple chemical sensitivity; other patients: FM/CFS-fibromyalgia/chronic fatigue syndrome).

Haplotype	Haplotype frequency (%)		
	MCS	SMCS	Other patients
CYP2C19 Ht*1/*2 - CYP2D6 Ht*1/*4 - AHR Arg554Lys	9.1	—	—
CYP2C19 Ht*1/*2 - CYP2D6 Ht*1/*4	5.5	1.1	6.25
CYP2C9 Ht*1/*2 - CYP2D6 Ht*1/*41	5.5	—	—
CYP2C9 Ht*1/*2 - CYP2D6 Ht*1/*4	3.6	6.4	—
CYP2C9 Ht*1/*3 - CYP2D6 Ht*1/*4	3.6	2.1	—
CYP2C9 Ht*1/*3 - CYP2D6 Ht*1/*41	3.6	—	—
CYP2C9 Ht*1/*3 - CYP2C19 Ht*1/*2 - CYP2D6 Ht*1/*4	1.8	—	—
CYP2C9 Ht*1/*3 - CYP2C19 Ht*1/*2 - CYP2D6 Ht*1/*4 - Arg554Lys	—	—	1.25
CYP2C9 Ht*1/*2 - CYP2C19*1/*2	1.8	—	—
CYP2C9 Ht*1/*2 - CYP2C19 Ht*1/*2 - AHR Arg554Lys	—	1.1	—
CYP2C9 Ht*1/*2 - CYP2C19 Ht*1/*2 - CYP2D6 PM*4/*4 - AHR Arg554Lys	—	—	1.25
CYP2C9 Ht*1/*3 - CYP2C19 Ht*1/*2	—	1.1	—
CYP2C9 Ht*1/*2 - CYP2D6 Ht*1/*41 - AHR Arg554Lys	—	1.1	—
CYP2C19 Ht*1/*2 - CYP2D6 Ht*1/*41	—	1.1	2.5
CYP2C9 Ht*1/*2 - CYP2D6 PM*4/*4	—	1.1	—
CYP2C9 Ht*1/*2 - CYP2D6 PM*4/*41	—	1.1	—
CYP2C9 Ht*1/*2 - CYP2D6 PM*41/*41 - Arg554Lys	—	—	1.25
CYP2C19 Ht*1/*2 - CYP2D6 PM*4/*4	—	1.1	1.25
CYP2C19 Ht*1/*2 - CYP2D6 PM*4/*4 - AHR Arg554Lys	—	—	1.25
CYP2C19 Ht*1/*2 - CYP2D6 PM*4/*41	—	2.1	—
CYP2C19 Ht*1/*2 - CYP2D6 PM*41/*41	—	1.1	—
CYP2C9 Ht*1/*3 - CYP2D6 PM*4/*41 - AHR Arg554Lys	—	—	1.25
CYP2C9 PM*2/*2 - CYP2D6 Ht*1/*41	1.8	—	—
CYP2C9 PM*3/*3 - CYP2C19 PM*2/*2	1.8	—	—
CYP2C9 PM*2/*2 - CYP2D6 PM*4/*41	—	1.1	—
CYP2C9 PM*3/*3 - CYP2D6 PM*41/*41	—	1.1	—
CYP2C19 PM*2/*2 - CYP2D6 Ht*1/*4	—	1.1	—
CYP2C19 PM*2/*2 - CYP2D6 Ht*1/*4 - AHR Arg554Lys	—	—	1.25
